# Inadvisable anti-vaccination sentiment: Human Papilloma Virus immunisation falsely under the microscope

**DOI:** 10.1038/s41541-017-0004-x

**Published:** 2017-03-08

**Authors:** Michael G. Head, Magdalen Wind-Mozley, Peter J. Flegg

**Affiliations:** 1 Faculty of Medicine, University of Southampton, Southampton, UK; 2 Independent researcher, Newbury, UK; 3 Department of Medicine, Blackpool Teaching Hospitals NHS Foundation Trust, Blackpool, UK

The Human Papilloma Virus (HPV) vaccine provides protection against the main cause of cervical cancer, and was licensed in both the US and the EU in 2006.^1^ As of October 2014, 64 countries and 12 overseas territories have implemented this vaccine into national immunisation schedules, with the vast majority of the coverage being in women in high-income settings.^2^ A 2016 review of 10 years of global HPV prevalence data has demonstrated reductions up to ~90% in HPV infections and genital warts, typically within four years after introduction of the vaccination programs, and across numerous countries on different continents.^3^ The impact of this vaccine is likely to be immense, with an estimated 345,000 cases of cervical cancer and 156,000 deaths predicted to be averted in 47 million women vaccinated by 2014 (ref. 2). The observed safety profile of the HPV vaccine has been excellent, with large well-conducted interventional and observational studies concluding there are no side-effects of significant prevalence that might counteract recommendations to implement the vaccine.^4–6^

During 2015 and 2016, case reports have been published of young women presenting with diverse physical and neurological symptoms following immunisation including symptoms similar to fibromyalgia, complex regional pain syndrome (CRPS) and postural orthostatic tachycardia syndrome (POTS).^7,8^ One case series of POTS was published in 2015 by the Danish Syncope Unit, headed by Dr Louise Brinth,^9^ and this resulted in Danish Health and Medicines Authority requesting that the European Medicines Agency (EMA) look into the safety aspects of the HPV vaccine. The EMA’s Pharmacovigilance Risk Assessment Committee has conducted a detailed and wide-ranging review of the evidence and published its findings in 2015 (ref. 10). The review highlighted the paucity of the quality of the studies making the claims (including three Brinth manuscripts), that prevalence of these conditions has not been shown to be significantly higher in vaccinated populations, and that a plausible biological mechanism by which the vaccination may induce CRPS and POTS has not been established. The conclusion was that the evidence did not support a causal link between HPV vaccines and CRPS or POTS.

However, in May 2016 Dr Brinth was co-signatory to a letter of complaint submitted to the EMA by individuals highlighting their affiliations with the Nordic Cochrane Centre.^11^ This letter, sent on Nordic Cochrane Centre-headed stationery despite not being an official Cochrane Collaboration communication, focused heavily on the conduct of the EMA review and issues such as conflict of interest, maladministration and confidentiality. However, as the EMA highlight in their detailed rebuttal,^12^ the authors ignored the limitations of the cited case reports and introduced basic errors such as accusing ‘the wrong Julie Williams’ of undeclared conflicts of interest.

Why does this matter? The Cochrane Collaboration has a long-held reputation of excellence, producing trusted high-quality reviews on thousands of topics related to health and has groups at national and sub-national level. The authors of the complaint, in using Cochrane-branded paper with the header ‘Trusted evidence. Informed decisions. Better health’, give the impression to readers that their views are representative of, or in some way approved by, the Cochrane Collaboration, and this is the view now being promoted in online anti-vaccine communities.^13,14^ We wrote to the governing Cochrane steering group raising this issue, and received a rapid reply from Professors Lisa Bero and Cynthia Farquhar, co-chairs of the steering group, who emphasised this is not an official Cochrane viewpoint and urged us to raise our concerns ‘in a public forum so they can be transparently discussed’.

There remains the ever-present risk that a vaccine scare will have an impact on uptake and, therefore, individual and population health. The government of Japan suspended its proactive recommendation of the HPV vaccine in June 2013, albeit continuing to make the vaccine available for those who request it. The decision was in response to unfounded fears about its safety profile and (as of 2016) they have not reversed their decision, despite the country’s own Vaccine Adverse Reactions Review Committee declaring the immunisation to be safe.^15^ Elsewhere, in circumstances not analogous to those outlined here, the UK knows only too well of the damage done due to the fabricated claims of Mr Andrew Wakefield and subsequent prolonged negative media coverage in relation to the MMR vaccine.^16^

It is imperative that the safety profiles of vaccinations continue to be rigorously considered so that emergence of linked health concerns can be identified and investigated. National and cross-national organisations such as the EMA are vital in monitoring the emerging data. However, we highlight here how academic colleagues, under the purported banner of a respected authority, raise concerns about the HPV vaccine but they cite an evidence base of small and poor quality studies and ignore the extensive wealth of global literature that vividly demonstrate the excellent efficacy and safety record of the vaccine. The infectious disease and oncology community should be aware of these claims and that they are not corroborated by the evidence base, and they must be able to communicate this to patients and the general public. As the EMA state, ‘the benefits of HPV vaccines, therefore, continue to outweigh their risks.’
